# The Asian Association for the Study of Diabetes Overview

**DOI:** 10.1111/jdi.12689

**Published:** 2017-05-05

## Executive Board Members

Chair: Yutaka Seino

Vice Chair: Hong‐Kyu Lee

Vice Chair: Wenying Yang

Editor in Chief for JDI: Nigishi Hotta

Directors:

Lee‐Ming Chuang
Nobuya Inagaki
Linong Ji
Weiping Jia
Takashi Kadowaki
Doo‐Man Kim
Moon‐Kyu Lee
Ronald Ma
Wayne H‐H Sheu
Yukio Tanizawa
Kathryn Tan
Zhiguang Zhou
Dalong Zhu

Auditors:

Seisuke Koumoto
Sung Rae Kim
Zhangrong Xu

## Affiliated Societies and Associations

Cambodian Diabetes Association

Chinese Diabetes Society

Chinese Taipei Diabetes Association

Diabetes Association of Thailand

Diabetes Hong Kong

Diabetes India

Diabetic Society of Singapore

Endocrine Society of Sri Lanka

Hong Kong Society of Endocrinology, Metabolism and Reproduction

Indonesian Diabetes Association

Japan Association for Diabetes Education and Care

Japan Diabetes Society

Korean Diabetes Association

Macau Diabetes Association

Diabetes Malaysia

Malaysian Diabetes Educators Society

Mongolian Diabetes Association

Myanmar Endocrinology and Metabolism Society

Philippine Diabetes Association

Taiwanese Association of Diabetes Educators

Vietnamese Association of Diabetes and Endocrinology

## Program Committee

Xueyao Han

Cheng Hu

Hung‐Yuan Li

Shih‐Yi Lin

Soo Lim

Ronald Ma

Eun‐Jung Rhee

Kathryn Tan

Daisuke Yabe

Hironori Waki

